# Risk Factors for Conversion from Laparoscopic to Open Appendectomy

**DOI:** 10.3390/jcm12134299

**Published:** 2023-06-27

**Authors:** Bruno Leonardo Bancke Laverde, Matthias Maak, Melanie Langheinrich, Stephan Kersting, Axel Denz, Christian Krautz, Georg F. Weber, Robert Grützmann, Maximilian Brunner

**Affiliations:** 1 Department of General and Visceral Surgery, Friedrich-Alexander-University, Krankenhausstraße 12, 91054 Erlangen, Germany; bruno.banckelaverde@uk-erlangen.de (B.L.B.L.); matthias.maak@uk-erlangen.de (M.M.); axel.denz@uk-erlangen.de (A.D.); christian.krautz@uk-erlangen.de (C.K.); georg.weber@uk-erlangen.de (G.F.W.); robert.gruetzmann@uk-erlangen.de (R.G.); 2 Department of General, Visceral, Thoracic and Vascular Surgery, University Greifswald, Ferdinand-Sauerbruch-Straße, 17475 Greifswald, Germany; melanie.langheinrich@med.uni-greifswald.de (M.L.); stephan.kersting@med.uni-greifswald.de (S.K.)

**Keywords:** acute appendicitis, laparoscopic approach, conversion, morbidity

## Abstract

(1) Background: Since its introduction in the 1990s, laparoscopic appendectomy has become established over the years and is today considered the standard therapy for acute appendicitis. In some cases, however, a conversion to the open approach is still necessary. The primary aim of this study was to identify risk factors for the need to convert from the laparoscopic to an open approach during appendectomy for acute appendicitis. (2) Methods: A retrospective analysis of 1220 adult patients who underwent laparoscopic appendectomy for acute appendicitis from 2010 to 2020 at the University Hospital Erlangen was performed. Data, including patient demographics and pre-, intra-, and postoperative findings, were collected and compared between patients with and without conversion. (3) Results: The conversion rate in our cohort was 5.5%. A higher preoperative WBC count and CRP (OR 1.9, *p* = 0.042, and OR 2.3, *p* = 0.019, respectively), as well as the presence of intraoperative perforation, necrosis or gangrene, perityphlitic abscess and peritonitis (OR 3.2, *p* = 0.001; OR 2.3, *p* = 0.023; OR 2.6, *p* = 0.006 and OR 2.0, *p* = 0.025, respectively) were identified as independent risk factors for conversion from the laparoscopic to the open approach. Conversion was again independently associated with higher morbidity (OR 2.2, *p* = 0.043). (4) Conclusion: The laparoscopic approach is feasible and safe in the majority of patients with acute appendicitis. Only increased inflammatory blood markers could be detected as the preoperative risk factors potentially influencing the choice of surgical approach but only with low specificity and sensitivity. For the decision to convert, intraoperative findings are additionally crucial. However, patients with conversion should receive special attention in the postoperative course, as these have an increased risk of developing complications.

## 1. Introduction

Acute appendicitis is one of the most common causes of emergency surgery. Current data on the incidence of acute appendicitis show persistently high values, which underlines the relevance of acute appendicitis in everyday surgical practice (151/100,000 person years in western Europe [1]; 123/100,000 person years in 2017 in Germany [2]). The laparoscopic approach for appendectomy was introduced in 1983 and has since gained in popularity. Today, the laparoscopic approach is considered the standard therapy [3,4].

The advantages of laparoscopic as compared to open appendectomy are shorter hospital stays, a decreased need for postoperative analgesia, early food tolerance and return to normal activities, and fewer postoperative complications [5,6,7]. However, these benefits are lost after the need for conversion to an open procedure [8,9]. In particular, the risk of surgical site infection increase significantly once conversion has taken place. In addition, an increased risk for other complications, such as intra-abdominal abscesses, appendiceal stump insufficiency, and bleeding, has been reported for patients requiring conversion during appendectomy [10,11]. Consequently, conversion is also associated with increased hospital costs and readmission rates [8].

Therefore, preoperative factors associated with a high risk of conversion becoming necessary might help in the decision regarding the optimal surgical approach for patients with acute appendicitis. Previous studies were already able to identify different parameters, including age, gender, diabetes, ASA Score, WBC and CRP values, the results of preoperative CT-scan, and the intraoperative presence of complicated appendicitis [8,12,13,14,15,16,17]. For example, Abe et al. were able to identify CT inflammation grade 4 or 5, complicated appendicitis, higher preoperative CRP level, and diffuse peritonitis as risk factors for conversion [14]; Gupta et al. showed that the conversion rate is highest in male patients above 45 years of age, with over 5 days’ duration of symptoms, leukocytosis of >20,000, and ruptured appendicitis on computed tomography scan [16]; and in the study of Antonacci et al., only the presence of comorbidities was independently associated with conversion [17].

The primary aim of this study is to identify risk factors for conversion from laparoscopic to open procedure. The secondary objective was to explore the consequence of conversion on postoperative morbidity.

## 2. Materials and Methods

This retrospective study was conducted on 1220 consecutive patients older than 18 years, who underwent laparoscopic appendectomy due to acute appendicitis at the department of general and visceral surgery of University Hospital of Erlangen between January 2010 and December 2020. Patients without intraoperatively or histopathologically acute appendicitis and those who underwent an appendectomy during other surgeries were excluded.

The following data were collected for analysis: patient demographics; preoperative laboratory and radiological data; and intraoperative details, including the level of training of the surgeon as well as postoperative outcome parameters, such as length of stay (LOS), postoperative complications, and readmission within 90 days postoperatively. Patients were divided into two groups (patients who did and did not require conversion) and recorded data were compared between the two groups to identify risk parameters for conversion.

This study was approved by the Ethics Committee of FAU Erlangen (22-157-Br).

### 2.1. Definitions

We defined complicated appendicitis according to the AAST classification as gangrenous or perforated appendicitis with or without peritonitis and/or perityphlitic abscess (AAST > II) [18]. This definition was applied on the one hand based on the intraoperative data (classified as intraoperatively assessed complicated appendicitis) and on the other hand based on the histological data (classified as histologically assessed complicated appendicitis). The interval between the first clinical examination and incision time is defined as the time to appendectomy. Morbidity was defined as any deviation from the normal postoperative course and was classified according to the Clavien–Dindo classification [19]. Morbidity ≥ grade III, according to Clavien–Dindo, was considered as major morbidity.

### 2.2. Diagnostic Algorithm

During the initial admission, all patients underwent a thorough clinical examination and a comprehensive blood test, encompassing a hemogram, kidney function, and inflammatory parameters. Moreover, a preoperative abdominal ultrasound was consistently conducted. In cases where the diagnosis remained uncertain, an abdominal CT scan was employed. Laparoscopy was indicated if there was confirmed appendicitis or a clinical suspicion based on the findings from all diagnostic procedures.

### 2.3. Surgical Procedure

Laparoscopic appendectomy was performed under general anesthesia using a three-port-technique. A 10–12 mm camera port was inserted periumbilically, two working ports (one 10–12 mm and one 5 mm) in the lower left quadrant and suprapubic or in the lower right quadrant. The appendix was removed using either an endo loop or an intestinal stapler. The intraoperative lavage of the abdomen was always performed. A drain was inserted when deemed appropriate by the surgeon.

Appendectomies were performed either by a specialist surgeon or by a resident. Residents were always assisted by a specialist surgeon who had experience in open and laparoscopic approaches and who decided to convert the procedure if necessary.

Open surgery was performed via infraumbilical median laparotomy or via Mc-Burney’s incision, depending on the decision of the specialist surgeon. The appendix was removed using a ligation of the appendiceal stump, a sinking of the appendiceal stump by purse-string suture, and an additional Z-suture. Again, the intraoperative lavage of the abdomen was always performed, and a drain was inserted when deemed appropriate by the surgeon.

### 2.4. Statistical Analysis

Statistical analyses were performed with SPSS Statistic (Version 22.0, IBM, Armonk, NY, USA). Data are presented as mean (range) or *n* (%). A Mann–Whitney U-test or a Student’s *t*-test was used to calculate and compare ordinal and metric data. The chi-square test was applied to categorical data. *p*-values of <0.05 were considered statistically significant. Multivariate analysis was performed with identified risk factors for conversion as well as for morbidity in univariate analysis. Parameters with incomplete data were excluded from multivariate analysis. The highest Youlden index, calculated through ROC analysis, was applied to determine the optimal cutoffs for metric risk factors included in the multivariate analysis. 

## 3. Results

### 3.1. Demographics

During the 11-year study period, 1220 patients underwent laparoscopic appendectomy for acute appendicitis. Of these, 67 (5.5%) patients had to be converted to open appendectomy. Table 1 summarizes patient demographics comparing laparoscopic and converted appendectomies. The conversion group was significantly associated with a higher age (52 vs. 38 years, *p* < 0.001), with a higher BMI (27.4 vs. 25.3 kg/m^2^, *p* = 0.013), with a worse ASA score (*p* < 0.001), and a higher rate of diabetes (9 vs. 4%, *p* = 0.040).

### 3.2. Preoperative, Intraoperative, and Histopathological Findings

Regarding the pre- and intraoperative as well as the histopathological data, thirteen factors differ significantly between the conversion and the no conversion group (Table 2): WBC count (14.6 vs. 12.8 × 10^9^/L, *p* = 0.034); CRP (170 vs. 56 mg/L, *p* < 0.001); hemoglobin level (13.6 vs. 14.3 g/dL, *p* = 0.008); the presence of intra-abdominal fluid in preoperative radiological diagnostic (51 vs. 33%, *p* = 0.004); surgical experience (*p* < 0.001); the duration of surgery 110 vs. 65 min, *p* < 0.001); the need for coecum resection (19 vs. 3%, *p* = 0.001); and the rate of perforation (67 vs. 17%, *p* < 0.001), of necrosis or gangrene (28 vs. 7%, *p* < 0.001), of perithyphilitic abscess (40 vs. 8%, *p* < 0.001), of peritonitis (52 vs. 23%, *p* < 0.001), and of intraoperatively complicated appendicitis (78 vs. 20%, *p* < 0.001) as well as of histopathologically complicated appendicitis (75 vs. 34%, *p* < 0.001).

### 3.3. Postoperative Outcomes

In our cohort, overall morbidity, major morbidity, and re-surgery rate were 5%, 3%, and 1% and the mean hospital stay was four days (standard deviation: +/− 3 days). The conversion group showed significantly higher morbidity (24 vs. 4%, *p* < 0.001); major morbidity (12 vs. 2%, *p* < 0.001); and a higher rate of wound infections (9 vs. 0%, *p* < 0.001), postoperative paralysis (4 vs. 0%, *p* < 0.001), and re-surgery (5 vs. 1%, *p* = 0.037) (Table 3). Moreover, patients with conversion needed a significantly longer hospital stay (8 vs. 3 days, *p* < 0.001) and more frequently required re-admission (9 vs. 2%, *p* = 0.003) (Table 3). One 80-year old patient with converted appendectomy died postoperatively due to a postoperative intestinal ischemia.

### 3.4. Multivariate Analysis of Risk Factors for Conversion

Multivariate analysis, including univariate identified risk factors for conversion, revealed two independent preoperative and four independent intraoperative risk factors (Table 4): a higher preoperative WBC count (OR 1.90 (1.02–3.53) *p* = 0.042); a higher preoperative CRP level (OR 2,27 (1.14–4.52 *p* = 0.019); and the presence of intraoperative perforation (OR 3.18 (1.59–6.38), *p* = 0.001), of intraoperative necrosis and/or gangrene (OR 2.29 (1.12–4.68), *p* = 0.023), of intraoperative perityphlitic abscess (OR 2,59 (1.32–5.08), *p* = 0.006), and of intraoperative peritonitis (OR 2.03 (1.09–3.79), *p* = 0.025).

### 3.5. Multivariate Analysis of Risk Factors for Morbidity

In a multivariate analysis for risk factors for postoperative morbidity, conversion could be identified as independent risk factor (OR 2.23 (1.03–4.85) *p* = 0.043) (Table 5). Next to the need of conversion, a higher age (OD 3.41 (1.80–6.46), *p* < 0.001), a higher preoperative WBC count (OR 2.15 (1.19–3.87), *p* = 0.011), a lower preoperative hemoglobin (OR 0.41 (0.22–0.77), *p* = 0.005) and a higher preoperative CRP value (OR 2.66 (1.38–5.13), *p* = 0.004) were further independent risk factors for morbidity (Table 5).

## 4. Discussion

The laparoscopic approach is today the absolute standard for performing an appendectomy for acute appendicitis. However, in some patients, a conversion to an open procedure is necessary. The knowledge of risk factors regarding the need for conversion—especially preoperative ones—could help with the decision of utilizing primarily an open approach for selected patients with a high conversion risk in order to avoid unnecessary costs, longer operating times, and an additional risk of morbidity.

In our present retrospective analysis of 1220 patients undergoing laparoscopic appendectomy, a conversion to open procedure was necessary in 67 cases (5.4%), which is consistent with previously published conversion rates [8,11,12,13,14,16,17,20].

Our study revealed six independent risk factors for conversion—two preoperative and four intraoperative ones: a higher preoperative WBC count; a higher preoperative CRP value; and the presence of an intraoperative perforation, of an intraoperative necrosis or gangrene, of an intraoperative perityphlitic abscess, and of an intraoperative peritonitis.

The identification of five of the six aforementioned risk factors is supported by previous studies [8,14,16,17,21]. Aydin et al. and Yigit et al. already demonstrated a significantly higher preoperative CRP level in patients who required conversion compared to those undergoing laparoscopic appendectomy, although the optimal cut off for a high CRP level varies slightly between the studies (≥108.5 mg/dL [12] and ≥119 mg/dL [15] vs. ≥95 mg/dL in our study). The level of the preoperative CRP may express the extent of appendicitis, which may be decisive for the decision to convert. Accordingly, the intraoperative signs of advanced appendicitis are again the decisive risk factors for the need for conversion. This is supported by our data, as patients with perforation, necrosis or gangrene, peritonitis and/or perithyphilitic abscess are more than twice as likely to convert. These intraoperative parameters have also already been reported in the literature [14,17]. The association of a higher preoperative WBC count with an increased conversion rate, which was detected in our study, has not been described in previous studies [12,15,16,21]. Similarly to an increased CRP, an increased WBC count may also represent the expression of a more advanced appendicitis, which makes this association appear plausible.

Age, diabetes, and ASA III-IV were further previously identified risk factors associated with a higher rate of conversion. Although these factors were also associated with an increased risk of conversion in the univariate analysis in our cohort, they failed to reach significance in the multivariate analysis. Another reported risk parameter for conversion, the male sex, could be confirmed as neither univariate nor multivariate in our analysis [8,12,13,16].

**Figure 1:**
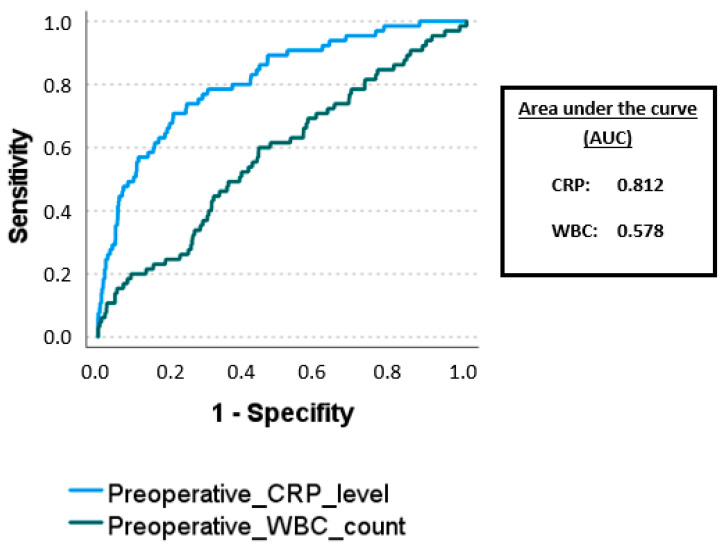
ROC analysis of preoperative WBC count and CRP level predicting the need of conversion.

As for the decision of the optimal surgical approach, only preoperative parameters can be considered, and preoperative WBC count and CRP level are the only possible decision criteria. However, both parameters have very limited specificity and sensitivity (WBC count of 13.4 × 10^9^/L: specificity 56%, sensitivity 60%, AUC 0.578; CRP level of 95 mg/L: specificity 80%, sensitivity 71%, AUC 0.812) (Figure 1). Based on these identified preoperative parameters, the decision to utilize an open approach may not be justified. Therefore, in our opinion, if there are no contraindications for laparoscopy, all patients with acute appendicitis should be laparoscopically explored.

The four identified intraoperative risk factors for conversion (the presence of an intraoperative perforation, of an intraoperative necrosis or gangrene, of an intraoperative perityphlitic abscess and of an intraoperative peritonitis), all representing the presence of a complicated appendicitis, are not suitable for the initial decision of surgical approach. However, a recent study has identified IL-6 as a promising biomarker predicting complicated appendicitis [22]. Therefore, IL-6 could potentially serve as an additional preoperative parameter, which was not investigated in our analysis.

In summary, all the identified risk factors represent parameters indicating an advanced and complicated appendicitis. Therefore, there is a certain degree of collinearity among all these risk factors. However, the data of our study suggest that the CRP level might be the most consistent parameter, as AUC of CRP showed the highest value.

However, our data show that once a conversion was required, patients were exposed to a significantly worse postoperative outcome—specifically, higher morbidity, higher wound infection, higher postoperative paralysis rate, and a higher rate of re-surgery, as well as longer postoperative hospital stays and a higher rates of readmissions. In addition, in multivariate analysis, conversion was confirmed as an independent risk factor for postoperative morbidity. These findings are in line with previous studies. A large Polish multicenter study showed a higher incidence of major morbidity in patients who underwent a conversion appendectomy [20]. Finnerty et al. reported a higher incidence of wound infection after conversion when compared with open and laparoscopic approaches [8]. Moreover, the literature confirms a higher rate of re-surgery and readmissions and a prolongated postoperative stay in patients requiring conversion [8,13,14,15]. In addition, although it was not investigated in our study, there are data from an American study that could reveal significantly higher costs in the group of converted patients compared the open or laparoscopic approaches [8]. Therefore, our data suggest that patients requiring conversion may benefit from special attention in the postoperative course and from a more customized therapy, as they are exposed to an increased postoperative risk. In addition, patients at risk could even benefit from an enhanced preoperative preparation, e.g., through an adequate preoperative re-hydration [23].

Further identified risk factors for postoperative morbidity in our analysis were a higher age, a higher preoperative WBC count and CRP level and a lower preoperative hemoglobin, which is in line with previous reports [24,25,26,27,28].

Several limitations exist regarding our data: First, the retrospective nature of this study and single-center design can incur some bias. Second, the reason for conversion was not investigated in our cohort, which may introduce some bias. Third, since appendectomies were performed by different surgeons, the conversion threshold always depends on individual judgement as well as the surgeon’s individual laparoscopic skills, which characterizes a potential bias.

## 5. Conclusions

Most cases of acute appendicitis can be successfully treated using the laparoscopic approach. The preoperative independent risk factors for the need for conversion (high preoperative WBC count and CRP level) identified in our study that could possibly influence the choice of surgical approach show a low specificity and sensitivity and, in our opinion, do not justify not primarily trying a laparoscopic approach. In addition to the preoperative risk factors, the decision to convert is also based on intraoperative findings. However, whenever conversion becomes necessary, there is an increased risk of postoperative complications, which should require increased attention in the postoperative course of the affected patients.

## Figures and Tables

**Table 1:** Baseline patient characteristics.

Patient Characteristics	No Conversion	Conversion	*p*-Value
**Number**	1153	67	
**Age (years)**	38 +/− 16	52 +/− 17	**<0.001**
**Gender** Female Male	566 (49) 587 (51)	35 (52) 32 (48)	0.706
BMI * (kg/m^2^)	25.3 +/− 5.0	27.4 +/− 6.6	**0.013**
ASA * I II III IV	676 (64) 346 (33) 42 (4) 0 (0)	17 (27) 30 (47) 15 (23) 2 (3)	**<0.001**
Smoker * No Former Current	649 (69) 51 (5) 237 (25)	36 (66) 3 (6) 16 (29)	0.861
**Diabetes**	41 (4)	6 (9)	**0.040**
**Crohn’s disease**	6 (1)	0 (0)	1.000
**Ulcerative colitis**	5 (0)	2 (3)	0.052

Data are presented as mean +/− SD or *n* (%). BMI = body mass index; ASA-score (ASA) = American Society of Anesthesiologists score; bold *p*-value = significant. * incomplete data: BMI: *n* = 873; ASA: *n* = 1128; smoker: *n* = 992.

**Table 2:** Preoperative, intraoperative, and histopathological findings.

	No Conversion (*n* = 1153)	Conversion (*n* = 67)	*p*-Value
**Preoperative blood results** WBC count (×10^9^/L) CRP (mg/L) Hemoglobin (g/dL) Creatinine (mg/dL)	12.8 +/− 4.3 56 +/− 67 14.3 +/− 1.5 0.8 +/− 0.3	14.6 +/− 6.5 170 +/− 124 13.6 +/− 1.9 0.9 +/− 0.3	**0.034** **<0.001** **0.008** 0.437
**Preoperative radiological diagnostic** Appendiceal diameter (mm) Intra-abdominal fluid Appendicolith	11 +/− 5 384 (33) 116 (10)	12 +/− 4 34 (51) 9 (13)	0.118 **0.004** 0.404
**Time to appendectomy** ≤6 h >6 h–≤12 h >12 h–≤24 h >24 h	470 (41) 359 (31) 223 (19) 101 (9)	24 (36) 22 (33) 11 (16) 10 (15)	0.335
**Surgical experience** Resident Specialist	376 (33) 777 (67)	19 (9) 61 (91)	**<0.001**
**Duration of surgery (min)**	65 +/− 24	110 +/− 42	**<0.001**
**Need of coecum resection**	43 (4)	13 (19)	**0.001**
**Intraoperative findings** Perforation Necrosis or gangrene Perithyphilitic abscess Intraoperatively assessed complicated appendicitis Peritonitis	193 (17) 85 (7) 88 (8) 227 (20) 262 (23)	45 (67) 19 (28) 27 (40) 52 (78) 35 (52)	**<0.001** **<0.001** **<0.001** **<0.001** **<0.001**
**Histopathological findings** Histopathologically assessed uncomplicated appendicitis Histopathologically assessed complicated appendicitis Malignancy	767 (67) 386 (33) 15 (1)	18 (27) 49 (73) 3 (5)	**<0.001** **<0.001** 0.067

Data are presented as mean (range) or *n* (%). WBC = white blood cell, CRP = C-reactive protein, bold *p*-value = significant.

**Table 3:** Postoperative outcome.

	No Conversion (*n* = 1153)	Conversion (*n* = 67)	*p*-Value
**Morbidity**	49 (4)	16 (24)	**<0.001**
**Cause for morbidity (*n* = 65)** **Surgical causes** Wound infection Intra-abdominal abscess Paralysis/ileus Intestinal perforation Bleeding Appendiceal stump insufficiency **Non-surgical causes** Cardio-pulmonal complication Uro-genital complication **Others**	4 (0) 9 (1) 1 (0) 3 (0) 2 (0) 0 (0) 4 (0) 5 (0) 21 (2)	6 (9) 1 (1) 3 (4) 0 (0) 0 (0) 0 (0) 1 (1) 0 (0) 5 (7)	-
**Major morbidity**	24 (2)	8 (12)	**<0.001**
**Clavien–Dindo** **I** **II** **III** **IV**	7 (1) 18 (2) 22 (2) 2 (0)	7 (10) 1 (2) 6 (9) 1 (2)	**<0.001**
**Mortality**	0 (0)	1 (2)	0.055
**Re-surgery**	11 (1)	3 (5)	**0.037**
**Length of postoperative hospital stay**	3 +/− 3	8 +/− 6	**<0.001**
**Readmission (within 90 days)**	21 (2)	6 (9)	**0.003**

Data are presented as mean (range) or *n* (%). Bold *p*-value = significant.

**Table 4:** Multivariate analysis of risk factors for conversion.

	Univariate	Multivariate		
	*p*-Value	OR	95% CI	*p*-Value
Age (high vs. low) *	**<0.001**	1.71	0.87–3.35	0.120
ASA (III/IV vs. I/II)	**<0.001**	2.11	0.87–5.09	0.098
Diabetes (yes vs. no)	**0.040**	1.08	0.33–3.49	0.900
Preoperative WBC count (high vs. low) *	**0.031**	**1.90**	**1.02–3.53**	**0.042**
Preoperative CRP (high vs. low) *	**<0.001**	**2.27**	**1.14–4.52**	**0.019**
Preoperative hemoglobin (high vs. low) *	**0.009**	0.60	0.32–1.13	0.110
Intra-abdominal fluid in radiological diagnostic (yes vs. no)	**0.004**	1.14	0.62–2.10	0.665
Coecum resection (yes vs. no)	**0.001**	1.76	0.77–4.02	0.182
Intraoperative perforation (yes vs. no)	**<0.001**	**3.18**	**1.59–6.38**	**0.001**
Intraoperative necrosis or gangrene (yes vs. no)	**<0.001**	**2.29**	**1.12–4.68**	**0.023**
Intraoperative perithyphilitic abscess (yes vs. no)	**<0.001**	**2.59**	**1.32–5.08**	**0.006**
Intraoperative peritonitis (yes vs. no)	**<0.001**	**2.03**	**1.09–3.79**	**0.025**

OR, odd ratio; CI, confidence interval. Duration of surgery and surgical experience (both as a consequence of conversion and of no risk factors) as well as BMI (missing data > 25%) were excluded. * Cut-offs for metric data assessed by ROC analysis: age: 40 years; WBC count: 13.4 × 10^9^/L; CRP: 95 mg/L; hemoglobin: 13.5 g/dL. Bold = significant.

**Table 5:** Multivariate analysis of risk factors for morbidity.

	Univariate	Multivariate		
	*p*-Value	OR	CI	*p*-Value
Age (high vs. low) *	**<0.001**	**3.41**	**1.80–6.46**	**<0.001**
Gender (female vs. male)	0.614	-	-	-
BMI (high vs. low) *	**0.010**	**	**	**
ASA (I/II vs. III/IV)	**<0.001**	1.76	0.80–3.90	0.161
Diabetes (yes vs. no)	0.107	-	-	-
Preoperative WBC count (high vs. low) *	**0.005**	**2.15**	**1.19–3.87**	**0.011**
Preoperative CRP (high vs. low) *	**<0.001**	**2.66**	**1.38–5.13**	**0.004**
Preoperative hemoglobin (high vs. low) *	**<0.001**	**0.41**	**0.22–0.77**	**0.005**
Preoperative creatinine (high vs. low) *	**0.007**	1.71	0.93–3.15	0.085
Intra-abdominal fluid in radiological diagnostic (yes vs. no)	**0.002**	1.60	0.89–2.85	0.114
Surgical experience (resident vs. specialist)	0.358	-	-	-
Coecum resection (yes vs. no)	**<0.001**	0.99	0.38–2.58	0.988
Conversion (yes vs. no)	**<0.001**	**2.23**	**1.03–4.85**	**0.043**
Intraoperative perforation (yes vs. no)	**<0.001**	1.38	0.68–2.79	0.375
Intraoperative necrosis or gangrene (yes vs. no)	**<0.001**	1.15	0.54–2.44	0.718
Intraoperative perithyphilitic abscess (yes vs. no)	**<0.001**	0.91	0.42–1.96	0.811
Intraoperative peritonitis (yes vs. no)	**0.001**	1.01	0.54–1.88	0.981

OR, odd ratio; CI, confidence interval. * Cut-offs for metric data assessed by ROC analysis: age: 50 years; BMI: 22.4 kg/m^2^; WBC count: 15.1 × 10^9^/L; CRP: 74 mg/L; hemoglobin: 13.6 g/dL; creatinine 0.9 mg/dL; ** excluded from multivariate analysis due to missing data >25%. Bold = significant.

## Data Availability

All data generated or analyzed during this study are included in this published article.
